# Endogenous angiotensins and catecholamines do not reduce skin blood flow or prevent hypotension in preterm piglets

**DOI:** 10.14814/phy2.12245

**Published:** 2014-12-23

**Authors:** Yvonne A. Eiby, Eugenie R. Lumbers, Michael P. Staunton, Layne L. Wright, Paul B. Colditz, Ian M.R. Wright, Barbara E. Lingwood

**Affiliations:** 1 UQ Centre for Clinical Research, The University of Queensland, Brisbane, Queensland, Australia; 2 School of Biomedical Sciences and Pharmacy, University of Newcastle, Newcastle, New South Wales, Australia; 3 Graduate School of Medicine and Illawarra Health and Medical Research Institute, University of Wollongong, Wollongong, New South Wales, Australia

**Keywords:** Blood pressure, cardiovascular, glucocorticoids, neonatal, skin blood flow

## Abstract

Endocrine control of cardiovascular function is probably immature in the preterm infant; thus, it may contribute to the relative ineffectiveness of current adrenergic treatments for preterm cardiovascular compromise. This study aimed to determine the cardiovascular and hormonal responses to stress in the preterm piglet. Piglets were delivered by cesarean section either preterm (97 of 115 days) or at term (113 days). An additional group of preterm piglets received maternal glucocorticoids as used clinically. Piglets were sedated and underwent hypoxia (4% FiO_2_ for 20 min) to stimulate a cardiovascular response. Arterial blood pressure, skin blood flow, heart rate and plasma levels of epinephrine, norepinephrine, angiotensin II (Ang II), angiotensin‐(1–7) (Ang‐(1‐7)), and cortisol were measured. Term piglets responded to hypoxia with vasoconstriction; preterm piglets had a lesser response. Preterm piglets had lower blood pressures throughout, with a delayed blood pressure response to the hypoxic stress compared with term piglets. This immature response occurred despite similar high levels of circulating catecholamines, and higher levels of Ang II compared with term animals. Prenatal exposure to glucocorticoids increased the ratio of Ang‐(1‐7):Ang II. Preterm piglets, in contrast to term piglets, had no increase in cortisol levels in response to hypoxia. Preterm piglets have immature physiological responses to a hypoxic stress but no deficit of circulating catecholamines. Reduced vasoconstriction in preterm piglets could result from vasodilator actions of Ang II. In glucocorticoid exposed preterm piglets, further inhibition of vasoconstriction may occur because of an increased conversion of Ang II to Ang‐(1‐7).

## Abstract (precis)

This study aimed to determine if immature hormonal control of the cardiovascular system contributes to preterm cardiovascular compromise. Physiological and hormonal responses of preterm piglets to hypoxia are immature compared with term piglets. This is not due to a lack of endogenous catecholamines or angiotensin II, but may be due to the differences in cardiovascular actions of the renin–angiotensin system.

## Introduction

Hypotension and low systemic blood flow are often present in very preterm neonates and are associated with an increased risk of disability and motor impairment (Osborn et al. 2007). These conditions are typically treated with sympathomimetic inotropes but the treatment failure rate is high at about 40% (Osborn et al. 2002). The underlying reasons for this cardiovascular compromise in the preterm infant are not fully understood. Various structural and physiological contributors to poor preterm *ex utero* cardiovascular function have been proposed with immature control of the cardiovascular system a key candidate.

At birth in many species, sympathetic neural control of the circulation is immature even at term (Mott 1969; Buckley et al. 1983; Gootman et al. 1992; Segar et al. 1998). If neural control is immature, then the endocrine system remains as the major controller of the cardiovascular system. In adults, three important hormonal systems are involved in cardiovascular control. The sympatho‐adrenal system plays a critical role in maintaining the adult circulation at rest and under stress via circulating catecholamines. The renin–angiotensin system plays a key role in controlling blood pressure primarily through the actions of angiotensin II (Ang II), a vasoconstrictor and a potent vasopressor. The hypothalamic–hypophyseal–cortisol axis has many actions including increasing the sensitivity to catecholamines (Noori et al. 2006). As well it increases myocardial calcium availability (Wehling 1994) and the activity of the renin–angiotensin system (Tangalakis et al. 1992).

However, these hormones could have different effects in neonates. In addition, their effects in preterm and term neonates could differ because hormone production, and therefore levels, are different. Receptor expression or sensitivity could also differ in preterm and term neonates. For example, the levels of expression of β_1_‐adrenoceptors in preterm heart is approximately half that of term heart (Kim et al. 2014a), and the number of α‐adrenoceptors in the vasculature is also reduced (Su et al. 1977; Shaul et al. 1990). The number of type 1 angiotensin receptors in the vasculature increases with gestation while the number of type 2 angiotensin receptors decreases (Burrell et al. 2001).

The aims of this study were to compare preterm and term piglets with respect to their (1) cardiovascular responses and (2) hormonal responses to a mild hypoxic stress; and to (3) determine if there is an association between these physiological and hormonal responses. We also aimed to identify the effects of maternal glucocorticoid exposure on preterm responses and associations.

## Materials and Methods

### Ethical approval

The project was approved by The University of Queensland Animal Ethics Committee (AEC Approval Number: UQCCR/999/08) and conforms to the Australian Code of Practice for the Care and Use of Animals for Scientific Purposes (8th edition 2013). All surgery was performed under general anesthesia, and all efforts were made to minimize suffering.

### Animals

Artificially inseminated pregnant sows (Large White × Landrace) were sourced from a commercial piggery owned by The University of Queensland at Gatton, Australia. All piglets were delivered by cesarean section either preterm at 97 days or term at 113 days (term ≈ 115 days). At 97 days of gestation, piglets are developmentally similar to a human infant born at approximately 27 weeks (Eiby et al. 2013). An additional group of preterm piglets were exposed to maternally administered glucocorticoids (betamethasone, 0.19 mg/kg body wt, i.m.; Celestone Chronodose; Schering‐Plough, Kenilworth, NJ) at 48 and 24 h prior to delivery. This dose/kg is equivalent to that given to women presenting with threatened preterm labor and has been demonstrated to mature the cardiovascular system of pigs (Eiby et al. 2012; Kim et al. 2014a). In each group, 12–15 piglets were randomly selected from 4 litters. Piglets weighing less than the 10th centile or greater than the 90th centile were excluded.

### Cesarean section

Pregnant sows (300–400 kg) were premedicated with 400 mg azaperone i.m. (Stresnil, Janssen, Australia) and 1000 mg ketamine i.m. (approximately 3 mg/kg; Ketamil; Troy Laboratories, Glendenning, NSW, Australia) and anesthesia induced with 200 mg of alfaxalone i.v.; (approximately 0.6 mg/kg; Alfaxan‐CD RTU; Jurox, Rutherford, NSW, Australia). Following intubation, anesthesia was maintained with 2% isoflurane (Attane Isoflurane USP; Minrad, International, Orchard Park, NY) in O_2_ with the sows breathing spontaneously.

Cesarean delivery was performed via a ventral midline incision followed by incision into the *linea alba* and then the uterine horn was partially exposed. Complete exposure of the uterine horns was avoided so that uterine blood flow was not compromised by stretching or occlusion of the uterine arteries. Piglets were removed from the uterus at varying time intervals (up to 20 min) and propofol (5 mg/kg i.v.) delivered to the fetus via the umbilical vein prior to clamping of the umbilical cord. After all piglets were delivered, the sow was euthanized (sodium pentobarbital 60 mL i.v.).

### Piglet intensive care and physiological monitoring

Details of piglet intensive care techniques have been reported elsewhere (Eiby et al. 2013). Briefly, all piglets were immediately intubated, ventilated, and sedated using a loading dose of morphine (200 μg/kg) and midazolam (100 μg/kg) followed by a maintenance infusion of morphine (40 μg/kg/h) and midazolam (120 μg/kg/h). This protocol is similar to the current sedation protocol used in preterm infants and has no cardio‐depressant effects in preterm or term infants (Quinn et al. 1992; Lemyre et al. 2004; Davies et al. 2008; Swart et al. 2012). Peak inspiratory pressure and FiO_2_ were adjusted to target pO_2_ 80–120 mmHg and pCO_2_ 35–45 mmHg. End expiratory pressure was maintained at 5–6 mmHg in all animals. A 3.5FG neonatal umbilical artery catheter (Argyle, Sherwood Medical, St. Louis, MO) was inserted into an umbilical artery for continuous measurement of arterial blood pressure (Transpac^®^ disposable pressure transducer; Hospira, Lake Forest, IL) and heart rate, and for collection of blood samples for immediate blood gas analysis (ABL815 Blood Gas Analyser; Radiometer^®^, Denmark) and for hormone analyses. Peripheral blood flow (skin blood flow) was monitored continuously with a Laser Doppler flowmetry surface probe (AD Instruments, Australia) attached over the gluteus muscle of the hind limb with an adhesive ring. Blood pressure and blood flow data were collected using a 16‐channel Powerlab and LabChart v7 software (AD Instruments, Bella Vista, NSW, Australia).

### Hypoxic stress

Following a stabilization period, pancuronium (0.1 mg/kg i.v.; AstraZenec, North Rhyde, NSW, Australia) was administered to prevent spontaneous breathing. This dose has no effect on cardiovascular parameters or plasma catecholamine levels in preterm babies, and the combined effects of pancuronium and morphine are very small (Quinn et al. 1992). At least 20 min later, hypoxia was induced by reducing the FiO_2_ to 4% for 20 min. This hypoxic regimen acts to stimulate a cardiovascular response but causes no or minimal neural injury at term (Lingwood et al. 2008; Harris et al. 2009). With the exception of two piglets where there were technical difficulties, piglets were studied between 3 and 5 h after birth with an average of 63 min between the first and last studied in each litter.

### Plasma hormone analyses

Plasma hormone levels of epinephrine, norepinephrine, Ang II, Ang‐(1‐7), and cortisol were measured at baseline and again at the end of hypoxia (20 min). At each time point, 2.5 mL of blood was collected of which 0.5 mL for catecholamine analysis was collected into a tube containing 5 mmol/L sodium metabisulphite and the remaining 2 mL into an EDTA tube. All samples were spun at ~1000 *g* for 10 min and then the plasma was frozen and stored at −80°C for later analyses. Plasma catecholamine levels were analyzed by Queensland Pathology at the Royal Brisbane and Women's Hospital (Brisbane, Australia), using reverse phase isocratic high‐performance liquid chromatography (HPLC) coupled with electrochemical detection (ECD). Sample purification was a single‐step procedure using alumina extraction. The method was linear to 500 nmol/L with a detection limit of 0.1 nmol/L and 95% precision. Plasma concentrations of Ang II, Ang‐(1‐7), and cortisol were measured by Prosearch Australia Pty. Ltd. (Malvern, Vic., Australia). Ang II and Ang‐(1‐7) were measured using a direct radioimmunoassay. Ang II assay sensitivity was 4 pmol/L; with intra‐ and interassay coefficients of variation of 6.4% and 12%, respectively. Ang‐(1‐7) assay sensitivity was 13 pmol/L; with intra‐ and interassay coefficients of variation of 4.5% and 10%, respectively. Cortisol was measured using a commercially available kit (Coat‐a‐Count^®^ kit; Siemens, Munich, Germany).

### Data analysis

Physiological measures, including mean arterial pressure, heart rate and skin blood flow, were averaged every 30 sec using LabChart 7 (AD Instruments). Baseline measures were obtained prior to hypoxia, when physiological parameters were stable. Skin blood flow was expressed as percentage of baseline value. Physiological data are presented as mean (SD in tables and ± SEM in figures) and hormonal data as median ± IQR.

Differences between groups in resting physiology and arterial blood gas parameters were identified using univariate ANOVAs (with group and sex as fixed factors and litter a random effect nested in group) and least squares difference post hoc analysis. Endocrine data were not normally distributed so were analyzed using nonparametric tests. Comparisons of hormone levels at baseline and at end hypoxic were analyzed using Kruskal–Wallis one‐way ANOVA with post hoc Mann–Whitney *U*‐tests to determine if groups differed and whether there was a sex difference within a group. Wilcoxon signed‐rank tests were used to determine if hormone levels changed within each group, both overall and by sex.

For 30‐sec epochs, mean blood pressure and skin blood flow were calculated for each animal and the data for all animals were correlated using Spearman correlations. This was done separately for each group at each 30‐sec interval throughout hypoxia. Spearman correlations were also used to determine the relationship between either mean arterial pressure or skin blood flow, and hormone levels within each group. To avoid reporting random type 1 errors, significant relationships were only reported where significance was found across consecutive time points spanning at least 4 min. All statistical analyses were conducted using IBM SPSS v21 (New York, USA) and the significance level used was 0.05.

## Results

### Piglet demographics and resting physiology

Both preterm groups had decreased body weight and baseline mean arterial pressures compared with the term group (Table 1, *P* < 0.05). Sex ratios were similar in each group and there was no effect of sex on any physiological or arterial blood gas parameters (Tables 1 and 2).

**Table 1.** Piglet demographic data and baseline physiology.

Variable	Preterm (*n* = 12–14)	Preterm +GC (*n* = 14–16)	Term (*n* = 13–15)
Body weight (g)*	1036 (211)	1043 (187)	1546 (322)
Sex ratio (M:F)	7:7	9:7	7:8
Mean arterial pressure (mmHg)*	31 (4)	30 (3)	39 (5)
Heart rate (bpm)	152 (26)	141 (15)	142 (19)

All values are mean (SD).

* Indicates that the term group is different to both the untreated preterm and preterm treated with maternal glucocorticoids (GC) groups.

**Table 2.** Blood gas parameters of piglets at baseline and end hypoxia.

Variable	Preterm (*n* = 7–14)	Preterm +GC (*n* = 7–16)	Term (*n* = 13–15)
PO_2_			
Baseline	183 (112)	111 (37)	115 (21)
End hypoxia	11 (2)	22 (9)	15 (1)
PCO_2_			
Baseline*	39 (9)	43 (6)	29 (7)
End hypoxia	70 (13)	57 (6)	47 (6)
pH			
Baseline*	7.49 (0.07)	7.45 (0.07)	7.59 (0.07)
End hypoxia	7.13 (0.07)	7.15 (0.06)	7.26 (0.04)
ABE			
Baseline	5.2 (2.0)	4.6 (2.2)	5.7 (2.1)
End hypoxia	−10.1 (2.0)	−9.4 (2.1)	−7.3 (0.2)

All values are mean (SD).

* Indicates that the term group is different to both preterm groups. There were no differences between groups at the end of hypoxia.

### Hypoxia

Blood gas parameters before and at the end of hypoxia are shown in Table 2. There were no significant differences in blood gas parameters between the groups at the end of hypoxia (Table 2). There were no correlations between baseline or end hypoxia pO_2_ or pCO_2_, and the physiological or hormonal responses to hypoxia. There was no effect of sex on any physiological or hormonal response to hypoxia and therefore combined data are presented.

### Physiological response to hypoxic stress

**Figure 1.**
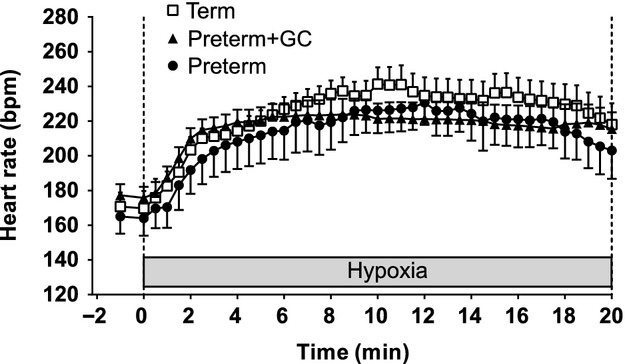
Heart rate responses to hypoxic stress (4% inspired oxygen) in untreated preterm piglets (closed circles), preterm piglets treated with maternal glucocorticoids (GC) (closed triangles), and term piglets (open squares). Data presented as mean (SEM) for 1 min of baseline followed by 20 min of hypoxia.

The heart rate response to the hypoxic stress was similar in all groups (Fig. 1). There was an increase to about 220 bpm at 5 min after the initiation of hypoxia that was maintained at this level for the duration of the hypoxic episode.

**Figure 2.**
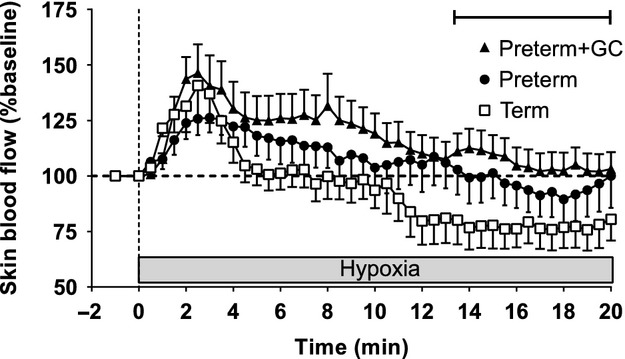
Skin blood flow responses to hypoxic stress (4% inspired oxygen) in untreated preterm piglets (closed circles), preterm piglets treated with maternal glucocorticoids (GC) (closed triangles), and term piglets (open squares). Data presented as mean (SEM) for 1 min of baseline followed by 20 min of hypoxia. The bar indicates a difference between the term group and the GC‐treated preterm group.

In term piglets, skin blood flow initially increased in response to hypoxia. This increase was followed after 5 min by a rapid decrease to baseline levels or below (Fig. 2). Preterm piglets had similar initial increases in skin blood flow but instead of decreasing, skin blood flow remained elevated so that in untreated preterm piglets, skin blood flow did not fall below baseline until 15 min after the onset of hypoxia. In glucocorticoid‐treated preterm piglets, skin blood flow remained elevated for longer. That is, skin blood flow in glucocorticoid‐treated preterm piglets did not fall below baseline at any time during hypoxia and was significantly higher than that measured in term piglets for most of the last 7 min of hypoxia (Fig. 2, *P* ≤ 0.05).

**Figure 3.**
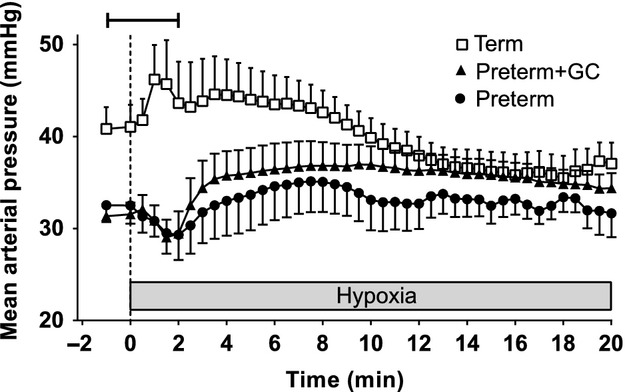
Blood pressure responses to hypoxic stress (4% inspired oxygen) in untreated preterm piglets (closed circles), preterm piglets treated with maternal glucocorticoids (GC) (closed triangles), and term piglets (open squares). Data presented as mean (SEM) for 1 min of baseline followed by 20 min of hypoxia. The bar indicates a difference between the term group and both preterm groups.

In response to hypoxia, term piglets had an immediate increase in mean arterial pressure that was maintained for about 9.5 min. This increase in blood pressure was delayed in both preterm groups. That is, there was an initial transient decrease in mean arterial pressure, after which blood pressure began to rise. This increase in blood pressure in preterm piglets did not occur until approximately 2 min after hypoxia began (Fig. 3).

**Figure 4.**
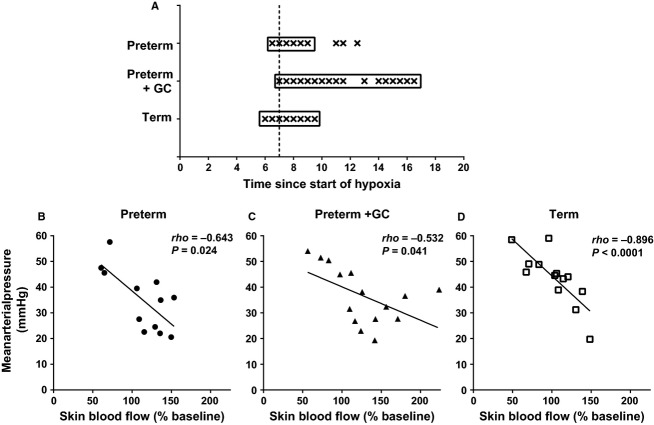
Skin blood flow and blood pressure. Panel A: In all groups, there were significant negative correlations between blood pressure and skin blood flow at multiple time points after the first 5 min of hypoxia as indicated by *. Actual relationships between blood pressure and skin blood flow in untreated preterm piglets (panel B), preterm piglets treated with maternal glucocorticoids (GC) (panel C), and term piglets (panel D) are shown at a representative time point indicated by the broken line in panel A.

In order to determine if the animals with the highest blood pressure were those with the greatest reduction in skin blood flow, blood pressure at each 30‐sec time point in every animal within each group was correlated against its skin blood flow at the same time point. This was done separately for all time points as this relationship may change across time. Only after the initial increase in blood pressure had occurred was mean arterial pressure negatively correlated with skin blood flow (Fig. 4, for all groups: *ρ* = −0.52 to −0.90, *P *<**0.05). The correlations were strongest in term piglets. This relationship continued until 10–17 min after the start of hypoxia.

### Hormonal response to hypoxic stress

#### Catecholamines

**Figure 5.**
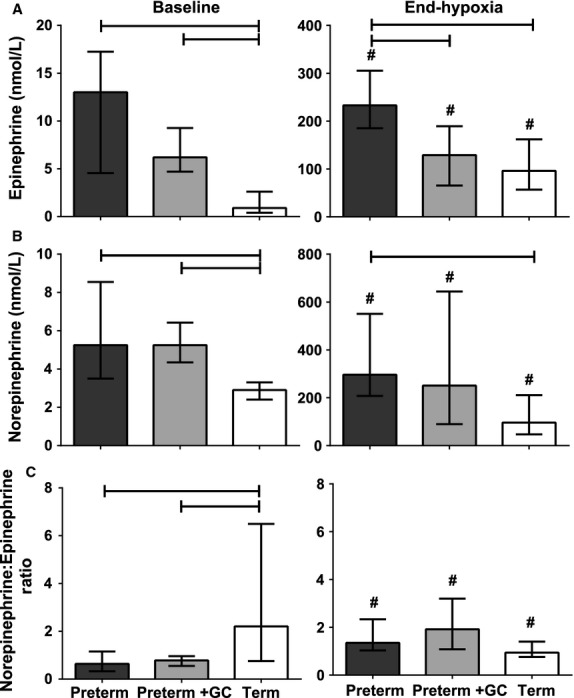
Plasma levels of (A) epinephrine, (B) norepinephrine, and (C) norepinephrine:epinephrine ratio during baseline and at the end of the hypoxic stress (4% inspired oxygen for 20 min) in preterm piglets, with and without maternal glucocorticoid treatment (GC), and term piglets. All values are median ± IQR. Note the different scales of the y axes. Bars indicate differences between groups. ^#^Indicates a difference between baseline and end hypoxia within a group.

Baseline norepinephrine and epinephrine levels were higher in both preterm groups than the term group (Fig. 5A and B, *P* < 0.01); there were no differences between the two preterm groups (*P *>**0.05). At the end of hypoxia, catecholamine levels in all three groups were about 40‐fold higher than baseline (Fig. 5A and B, *P* < 0.05), and epinephrine and norepinephrine levels in untreated preterm piglets were higher than in term animals (*P *<**0.05). Levels in glucocorticoid‐exposed preterm piglets were similar to those in term piglets. The baseline norepinephrine:epinephrine ratio was lower in both preterm groups compared with the term group (Fig. 5C, *P* < 0.05). At the end of hypoxia, this ratio had increased in both the untreated preterm (*P *=**0.008) and glucocorticoid‐treated preterm (*P *=**0.039) groups, but decreased in the term group (*P *=**0.036) so that there was no longer any difference between term and preterm groups.

#### Cortisol

**Figure 6.**
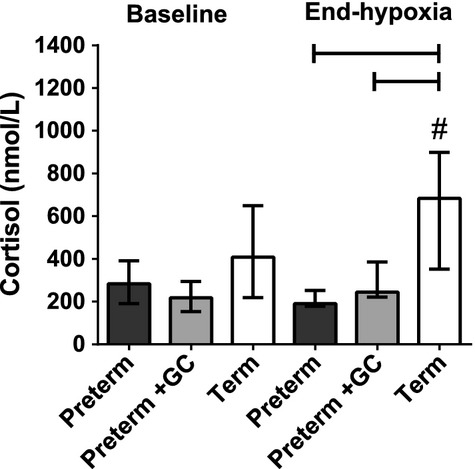
Plasma cortisol levels at baseline and at the end of the hypoxic stress (4% inspired oxygen for 20 min) in preterm piglets, with and without maternal glucocorticoid (GC) treatment, and term piglets. All values are median ± IQR. Bars indicate differences between groups. ^#^Indicates a difference between baseline and end hypoxia within a group.

Baseline cortisol levels were similar in the three groups (Fig. 6, *P* = 0.103). In term piglets, cortisol levels increased in response to hypoxia (Fig. 6, *P* = 0.028). There was no rise in cortisol levels in either group of preterm piglets in response to the hypoxic stress.

#### Angiotensins

**Figure 7.**
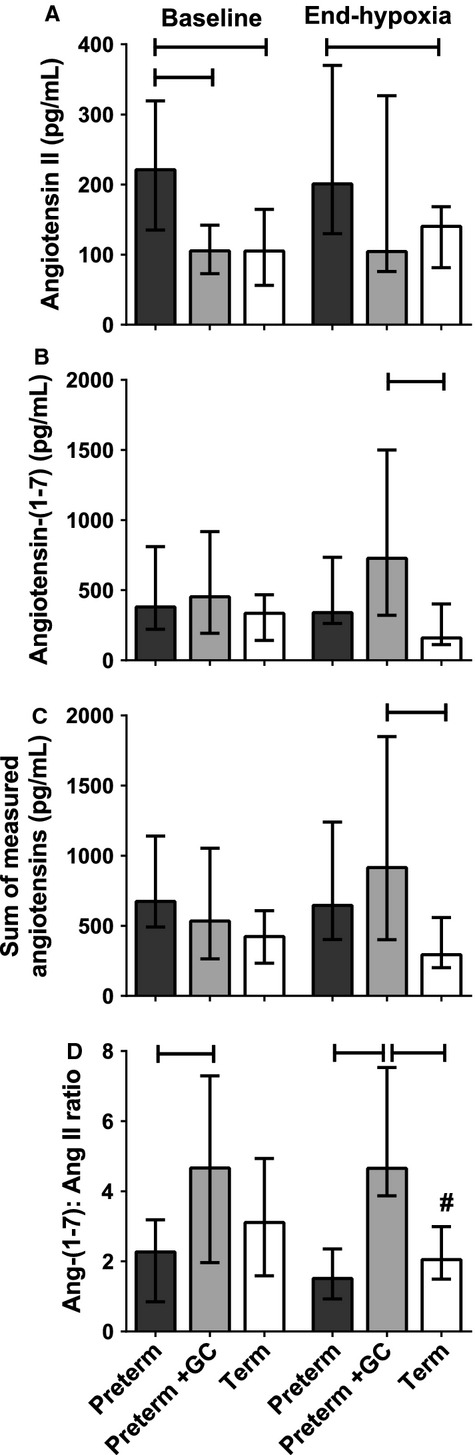
Plasma levels of (A) Angiotensin II, (B) Angiotensin‐(1‐7), (C) sum of measured angiotensin peptides (D) Angiotensin‐(1‐7): Angiotensin II ratio at baseline and at the end of the hypoxic stress (4% inspired oxygen for 20 min) in preterm piglets, with and without maternal glucocorticoid (GC) treatment, and term piglets. All values are median ± IQR. Bars indicate differences between groups. ^#^Indicates a difference between baseline and end hypoxia within a group.

Baseline Ang II levels were significantly higher in untreated preterm piglets compared with term piglets (Fig. 7A; *P *=**0.001), but levels in glucocorticoid‐exposed preterm piglets were lower than untreated preterm piglets (*P *=**0.001) and not different from term piglets (*P *=**0.71). There was no effect of hypoxia on Ang II levels in any group. At the end of hypoxia, therefore, Ang II levels were still higher in untreated preterm animals compared with term animals (*P *=**0.007) and the mean level in glucocorticoid‐exposed preterm piglets was lower than in untreated preterm animals, although this did not quite reach significance (*P *=**0.053). There was no difference in Ang II levels between glucocorticoid‐exposed preterm and term groups after hypoxia (*P* = 0.68).

The three groups of piglets had similar baseline levels of Ang‐(1‐7) (Fig. 7B, *P* > 0.05). However, at the end of hypoxia, Ang‐(1‐7) levels were higher in the glucocorticoid‐treated preterm group compared with the term group (*P *=**0.006). Untreated preterm animals also tended to have higher levels than term animals (*P *=**0.058). Ang‐(1‐7) levels at the end of hypoxia were not different from baseline.

Ang‐(1‐7) is primarily formed by conversion from Ang II. Therefore, increases in Ang‐(1‐7) levels could result from increased secretion of Ang II or increased conversion of Ang II to Ang‐(1‐7) or both. We calculated the sum of the measured angiotensin peptides (i.e., the sum of Ang II and Ang‐(1‐7) levels) as a measure of increased production of these two peptides. There were similar values in the three groups at baseline but at the end of the hypoxic period, levels were higher in the glucocorticoid‐exposed preterm group compared with the term group (Fig. 7C, *P* = 0.029). There was no overall effect of hypoxia on the sum of measured angiotensin peptides.

Increased conversion of Ang II to Ang‐(1‐7) would be reflected in the ratio of Ang‐(1‐7):Ang II. The Ang‐(1‐7):Ang II ratio at baseline was significantly higher in glucocorticoid exposed preterm piglets compared with untreated preterm piglets (Fig. 7D, *P* = 0.015). At the end of hypoxia, the Ang‐(1‐7):Ang II ratio in the glucocorticoid exposed preterm group was higher than in both other groups (*P *=**0.001 compared to untreated preterm and *P *<**0.01 compared to term). The Ang‐(1‐7):Ang II ratio was reduced following hypoxia only in the term group (*P *=**0.036).

### Is there an association between these physiological and hormonal responses?

To determine if hormone levels may contribute to changes in blood pressure or skin blood flow, we used correlation analysis to identify associations between physiological variables and hormone levels. For all animals within a group, blood pressure or skin blood flow was correlated against either baseline or end‐hypoxia hormone levels. This was done separately for each 1‐min physiological measurement.

Using this method we found that, at multiple time points over the first 7–10 min of hypoxia, mean arterial pressure was positively correlated with end‐hypoxia Ang II and Ang‐(1‐7) levels in term animals only (*ρ* = 0.57–0.79, *P *=**0.004–0.045). In this group, mean arterial pressure from 4 to 15 min after the onset of hypoxia was also positively correlated with baseline cortisol levels (*ρ* = 0.41–0.73, *P *=**0.011–0.043). In glucocorticoid‐exposed preterm piglets, there was a positive correlation between mean arterial pressure over the first 12 min of hypoxia and end‐hypoxia norepinephrine levels (*ρ* = 0.63–0.82, *P *=**0.001–0.007). This was not found in the other groups.

**Figure 8.**
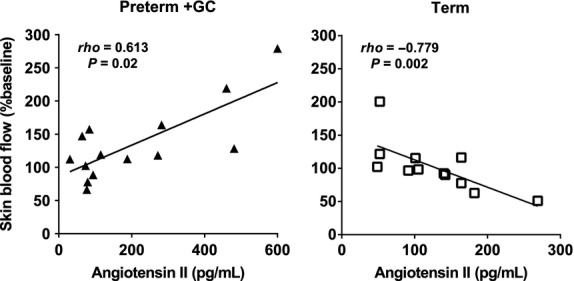
The relationship between skin blood flow at 8 min after the commencement of hypoxia and angiotensin II levels at the end of hypoxia for preterm piglets treated with maternal glucocorticoids (GC) and term piglets. Note the different scales of the x axis.

In term piglets only, skin blood flow was negatively correlated with Ang II (end hypoxia) (Fig. 8) and Ang‐(1‐7) (baseline and end hypoxia) from 7 to 20 min after commencing hypoxia (*ρ* = −0.52 to −0.86, *P *=**0.002–0.045), and with baseline cortisol levels from 12 to 19 min of hypoxia (*ρ* = −0.63 to −0.81, *P *=**0.003–0.039). By contrast, in the glucocorticoid‐treated preterm group, skin blood flow at 3–14 min into hypoxia was positively correlated with Ang II levels (end hypoxia) (Fig. 8) (*ρ* = 0.57–0.66, *P *=**0.01–0.034). There were no correlations between skin blood flow and hormone levels in untreated preterm piglets.

## Discussion

The physiological and hormonal responses of the preterm piglet to a hypoxic stress are different from those of the term piglet. *Ex utero* term piglets and lambs, and human infants respond to an hypoxic stress with an increase in heart rate and blood pressure, and a reduction in skin blood flow as blood is diverted from the periphery to maintain essential organ blood flow (Harris et al. 2009; Cheung et al. 2011; Cohen et al. 2012). Preterm piglets do not mount this mature response to hypoxic stress. The preterm piglet has limited peripheral vasoconstriction in response to hypoxia, and may have a limited capacity to increase cardiac output. Our results indicate that this immature response is not due to a lack of endogenous catecholamines or angiotensin II, but may be due to the alterations in the cardiovascular actions of the renin–angiotensin system.

The physiological differences between preterm and term piglets at baseline are similar to those observed in preterm and term human infants. Blood pressure is lower in preterm human infants compared with term infants and it was lower in both untreated and glucocorticoid‐exposed preterm piglets (Kluckow and Evans 1996).

During hypoxia all groups had similar initial heart rate and skin blood flow responses. The tachycardia is probably not the result of increased cardiac sympathetic nerve activity because the sympathetic nervous system is immature in preterm piglets (Buckley et al. 1983; Gootman et al. 1992). Instead, it could be due to increased levels of circulating catecholamines, or withdrawal of cardiac vagal tone as this pathway is mature prior to term (Lumbers et al. 1979). In the absence of other changes, the tachycardia would cause a rise in cardiac output in all three groups of piglets. The increase in skin blood flow in the first few minutes of hypoxia both in term and preterm piglets could be a local tissue response to hypoxia or a passive response to an increase in cardiac output.

In light of these very similar initial heart rate and skin blood flow responses in term and preterm piglets in the first minutes of hypoxia, it is perhaps surprising that preterm piglets had a different blood pressure response compared with term piglets. In term piglets, there was a sharp increase in blood pressure despite the increased skin blood flow and consequent reduction in peripheral vascular resistance. This suggests that there was an increase in cardiac output sufficient to counteract the effects of a reduction in peripheral vascular resistance on blood pressure. In contrast, in preterm piglets, any increase in cardiac output that may have resulted from the tachycardia was outweighed by the decrease in vascular resistance so that blood pressure decreased rather than increased in the first few minutes of hypoxia. It is likely that the preterm heart is not able to increase contractility to the same degree as the term heart and this may result in a smaller increase in cardiac output in preterm piglets compared to term piglets. This may be due to the immaturity of the myocytes with a lower density of myofilaments and a poorly developed sarcoplasmic reticulum (Smolich 1995). It could also be the result of a lower density of β_1_‐adrenoceptors in the ventricles leading to a poor response to endogenous catecholamines (Kim et al. 2014a).

As hypoxia progressed, there was a sharp fall in skin blood flow in term piglets. The significant negative correlation between blood pressure and skin blood flow at this time suggests that vasoconstriction contributes to the maintenance of elevated blood pressure until the heart begins to fail from the effects of hypoxia 8–10 min after the initiation of hypoxia. There was a lesser reduction in skin blood flow in preterm piglets and it occurred later. This vasoconstriction, together with the sustained increase in heart rate probably drove the delayed rise in blood pressure, although the pressure never reaches that seen in the term piglets. The sustained nature of this pressor response is congruent with the known greater hypoxia resistance of the preterm piglets (Singer 1999; Wassink et al. 2007).

The reduction in skin blood flow was least in the glucocorticoid‐exposed preterm piglets. In this group, skin blood flow did not fall below baseline, in contrast to untreated preterm piglets where skin blood flow did fall below baseline levels by the end of hypoxia. Vasodilation and an inability to shutdown skin blood flow appear to be a feature of preterm physiology, as high skin blood flow occurs in preterm babies and is associated with increased illness severity and mortality (Stark et al. 2008a,b). Despite this poor vascular response to hypoxic stress, blood pressure in glucocorticoid‐exposed preterm piglets was at least as high as untreated preterm piglets suggesting an improved capacity to increase cardiac contractility. This may be due to increased cardiac structural maturity (Kim et al. 2014a) and/or increased cardiac β_1_‐adrenoceptor expression (Kim et al. 2014b) following glucocorticoid exposure.

In summary, our results suggest that the preterm piglet has limited capacity to increase cardiac output in response to hypoxia, possibly due to a limited increase in cardiac contractility. In the preterm piglet, the capacity to reduce skin blood flow was also limited and this was most marked in glucocorticoid‐exposed preterm animals.

Could these differences between preterm and term piglets be the result of differences in hormonal control of the cardiovascular system? The lower blood pressure of preterm piglets and their limited capacity to reduce skin blood flow during hypoxia is not due to a lack of circulating catecholamines. Epinephrine and norepinephrine levels in preterm piglets were higher than in term piglets and increased markedly during the hypoxic stress. Preterm piglets produce catecholamines in abundance both at baseline and in response to stress. Although previous studies report variable levels of catecholamines in preterm infants (Lagercrantz and Bistoletti 1977; Newnham et al. 1984; Greenough et al. 1987; Ekblad et al. 1992; Mehandru et al. 1993), all agree that at the time of birth, preterm infants can release catecholamines. There is also agreement that the preterm sympathetic nervous system is immature. Norepinephrine is thought to reflect neuronal spill over in addition to adrenal medullary secretion while epinephrine represents only adrenal release (Ervin et al. 2000). Thus, the lower norepinephrine:epinephrine ratio at baseline in the preterm piglet indicates reduced sympathetic nervous system activity compared to term piglets, and agrees with previous findings that the sympathetic system is immature prior to term (Buckley et al. 1983; Gootman et al. 1992; Segar et al. 1998; Yu and Lumbers 2000). Following hypoxia, the ratio is similar in all groups suggesting that all groups are similarly dependent on circulating catecholamines during hypoxia (Dong et al. 2011).

Despite high levels of catecholamines in preterm piglets, blood pressures were low and the peripheral vascular resistance stayed low, suggesting that catecholamines do not affect cardiovascular function in the same way as in the adult. Thus, there were few significant associations between catecholamine levels and blood pressure or skin blood flow. The only significant correlation was in the glucocorticoid‐exposed preterm group where there was a positive correlation between mean arterial blood pressure early in hypoxia and norepinephrine levels during hypoxia. This suggests a maturational effect of glucocorticoids on the sympathetic nervous system as seen in fetal lambs (Segar et al. 1998), so that this system is now influencing blood pressure.

Several factors may explain why the preterm piglet is unresponsive to catecholamines. The preterm heart has fewer β_1_‐adrenoceptors potentially limiting inotropic action of catecholamines (Chen et al. 1979; Hatjis and McLaughlin 1982; Kim et al. 2014b). Also, the preterm heart is structurally immature (Smolich 1995; Rudolph 2000; Kim et al. 2014a) so it may be unable to increase contractility despite high levels of catecholamines. Vasoconstrictor effects may also be limited. The low ratio of norepinephrine to epinephrine at baseline in preterm animals (Fig. 5C) may contribute to reduced vasoconstriction. Epinephrine is a much more effective β‐adrenoceptor agonist than norepinephrine which has more pronounced α‐adrenoceptor‐mediated effects. This means that epinephrine‐mediated vasodilatation (via peripheral β_2_‐adrenoceptors) may outweigh norepinephrine‐mediated vasoconstriction. However, despite the increased ratio of norepinephrine to epinephrine during hypoxia, preterm piglets still did not vasoconstrict as effectively as term piglets. A reduced response to norepinephrine may result from a reduced number of α‐adrenoceptors in the peripheral blood vessels of preterm piglets compared with term piglets (Su et al. 1977; Shaul et al. 1990).

A reduced response to infused catecholamines has been reported in preterm infants where many babies show limited response to dopamine or dobutamine administered to support cardiovascular function (Osborn et al. 2002; Ishiguro et al. 2012).

Preterm piglets, in contrast to term animals, did not increase cortisol levels in response to the hypoxic stress. Many preterm infants also have a reduced cortisol response to stress and low cortisol levels have been associated with hypotension (Scott and Watterberg 1995; Ng et al. 2004; Fernandez and Watterberg 2009). However, cortisol levels during hypoxia were not correlated with the physiological response to hypoxia, suggesting that lack of cortisol was not the direct cause of the altered preterm response. It is likely that cortisol has indirect effects that improve cardiovascular function via maturation of various systems. The positive correlation between baseline cortisol levels and blood pressure and the negative correlation between baseline cortisol levels and skin blood flow in the term piglets suggests that animals with increased cortisol secretion before hypoxia, perhaps reflecting a greater maturity, are those with the strongest response. There is evidence in fetal sheep that elevated cortisol improves blood pressure by increased sensitivity to Ang II (Tangalakis et al. 1992).

The altered response of preterm piglets to hypoxia may also be due to altered actions of the renin–angiotensin system. Untreated preterm piglets had higher baseline levels of Ang II compared with term piglets. This has also been reported for human preterm infants (Miyawaki et al. 2006). Despite high Ang II levels, preterm piglets had lower blood pressures than term piglets. Although Ang II is a potent vasopressor, its pressor effect in the adult relies on actions within the central nervous system that increase sympathetic outflow to peripheral vessels (Scroop and Whelan 1968; Lumbers et al. 1979). The sympathetic nervous system begins to influence cardiovascular function around the time of birth in the piglet (Buckley et al. 1983; Gootman et al. 1992; Yu and Lumbers 2000), and thus these sympathetically mediated actions of Ang II could account for the positive correlation between blood pressure and Ang II levels and the negative correlation between skin blood flow and Ang II levels seen in term piglets only.

If sympathetic innervation of the vasculature is immature, as is the case in the preterm piglet and probably the preterm human (Buckley et al. 1983; Gootman et al. 1992; Yu and Lumbers 2000), the only vasoconstrictor action of Ang II would be a direct effect via the angiotensin type 1 receptor (AT_1_R), and even in the adult human this direct vasoconstrictor action of Ang II is weak (Scroop and Whelan 1968). But Ang II can also act via angiotensin type 2 receptors (AT_2_R) and have a vasodilator effect. In the preterm fetal sheep systemic vasculature, AT_2_Rs are more numerous than AT_1_Rs with the ratio reversing as term approaches (Burrell et al. 2001). We have made similar observations in piglet kidney (B. E. Lingwood, unpublished observations). Thus, in preterm vasculature, which lacks sympathetic innervation, and in which AT_2_Rs outnumber AT_1_Rs, the dominant action of Ang II is likely to be vasodilator rather than vasoconstrictor. In vitro studies in fetal sheep are consistent with this hypothesis. Ang II causes only a transient vasoconstriction followed by relaxation in renal and mesenteric artery, where AT_2_Rs are probably dominant, but a sustained contraction in umbilical artery (absent in the preterm neonate), where AT_2_Rs are almost absent (Burrell et al. 2001; Segar et al. 2001). In the preterm, this could explain the lower blood pressure, the limited vasoconstrictor response to hypoxia, and the switch from negative to positive correlation between skin blood flow and Ang II levels in glucocorticoid‐exposed preterm piglets. The lack of change in Ang II levels with hypoxia is consistent with observations in fetal sheep (Ervin et al. 2000).

Interestingly, exposure to glucocorticoids was associated with lower baseline Ang II levels than those found in untreated preterms. A similar situation has been reported in fetal sheep (Ervin et al. 2000). This may be due to a increased conversion of Ang II to Ang‐(1‐7), resulting in increased Ang‐(1‐7) levels and the increased Ang‐(1‐7):Ang II ratio in glucocorticoid‐exposed piglets, particularly following hypoxia. As Ang‐(1‐7) is also vasodilator at both the AT_2_R and Mas receptor (Hilliard et al. 2013), this pathway could also contribute to the higher skin blood flow observed in glucocorticoid‐exposed preterm piglets during hypoxia.

We did not detect any differences between male and female piglets that might explain the higher microvascular flow and worse outcomes for male preterm infants (Stark et al. 2008a; Kent et al. 2012). A previous animal study demonstrating sex effects in vascular function following hypoxia included more than 60 animals of each sex (Bennet et al. 2007), and thus our study may have had insufficient power to detect relatively small sex differences. A possible limitation of the study is the use of hormone levels at the end of hypoxia rather than measuring them serially throughout the hypoxic episode but this would have required multiple blood samples during hypoxia which would induce hypovolemia and associated confounding factors. We have previously observed a very strong correlation between hormone levels at the end of hypoxia and levels earlier in hypoxia (B. L. Lingwood, unpublished data). We cannot rule out the possible influence of other vasoactive factors such as vasopressin, nitric oxide, and endothelium‐derived hyperpolarizing factor. Results are unlikely to be confounded by the use of sedation. Morphine at a similar dose to that used in this study has no cardio‐depressant effects in preterm or term infants (Quinn et al. 1992; Lemyre et al. 2004).

A greater understanding of the receptors and actions of the renin–angiotensin system in the preterm neonate and their role in preterm cardiovascular compromise will likely open new targets for effective therapies.

### Perspectives

In conclusion, aspects of preterm cardiovascular physiology are immature, but there is no lack of circulating catecholamines or angiotensins. Glucocorticoid treatment does not improve the cardiovascular response to hypoxia. Immature receptor profiles within the renin–angiotensin system, and an additive effect of glucocorticoids leading to increased conversion of Ang II to Ang‐(1‐7), may contribute to an inability to appropriately limit peripheral blood flow to maintain vital organ blood flow in the preterm neonate. Our findings suggest that inotropes that target the adrenergic system may be of limited effectiveness in clinical practice. We suggest that limiting potential detrimental effects of the renin–angiotensin system will yield better treatments for preterm cardiovascular compromise.

## Acknowledgments

The authors wish to recognize the important role of the large team involved in the intensive care of the piglets, particularly Sonia Sam and of the veterinary team primarily involved in the care of the sow and the cesarean section, Drs Helen Keates and Ranald Cameron.

## Conflict of Interest

None declared.
